# Gene transfer between fungal species triggers repeated coffee wilt disease outbreaks

**DOI:** 10.1371/journal.pbio.3002901

**Published:** 2024-12-06

**Authors:** Brenda D. Wingfield, Michael J. Wingfield

**Affiliations:** Department of Biochemistry, Genetics and Microbiology, Forestry and Agricultural Biotechnology Institute, University of Pretoria, Pretoria, South Africa

## Abstract

Two outbreaks of coffee wilt disease have devastated African coffee production. A PLOS Biology study suggests that horizontal gene transfer via Starships between two fungal strains played a key role in the repeated emergence of the disease.

## Teaser

Two outbreaks of coffee wilt disease have devastated African coffee production. A *PLOS Biology* study suggests that horizontal gene transfer via large Starship transposons between 2 fungal species played a key role in the repeated emergence of the disease.

Coffee is one of the most popular beverages globally, produced from the fruit of several species in the tree genus *Coffea*. Among the diseases affecting coffee, coffee wilt disease (CWD), caused by the fungus *Fusarium xylarioides* has received surprisingly little attention, at least in comparison to coffee rust and coffee berry disease. This is partly due to confusion surrounding the identification of the causal agent [1] and a fragmented knowledge of past outbreaks. Until the mid-1900s, coffee production in Africa was predominantly based on *Coffea excelsa* and *Coffea arabica*. However, the devastating impact of CWD led to the replacement of *C*. *excelsa* by *C*. *canephora* (also known as *C*. *robusta*) in many regions of the continent [2].

**Fig 1:**
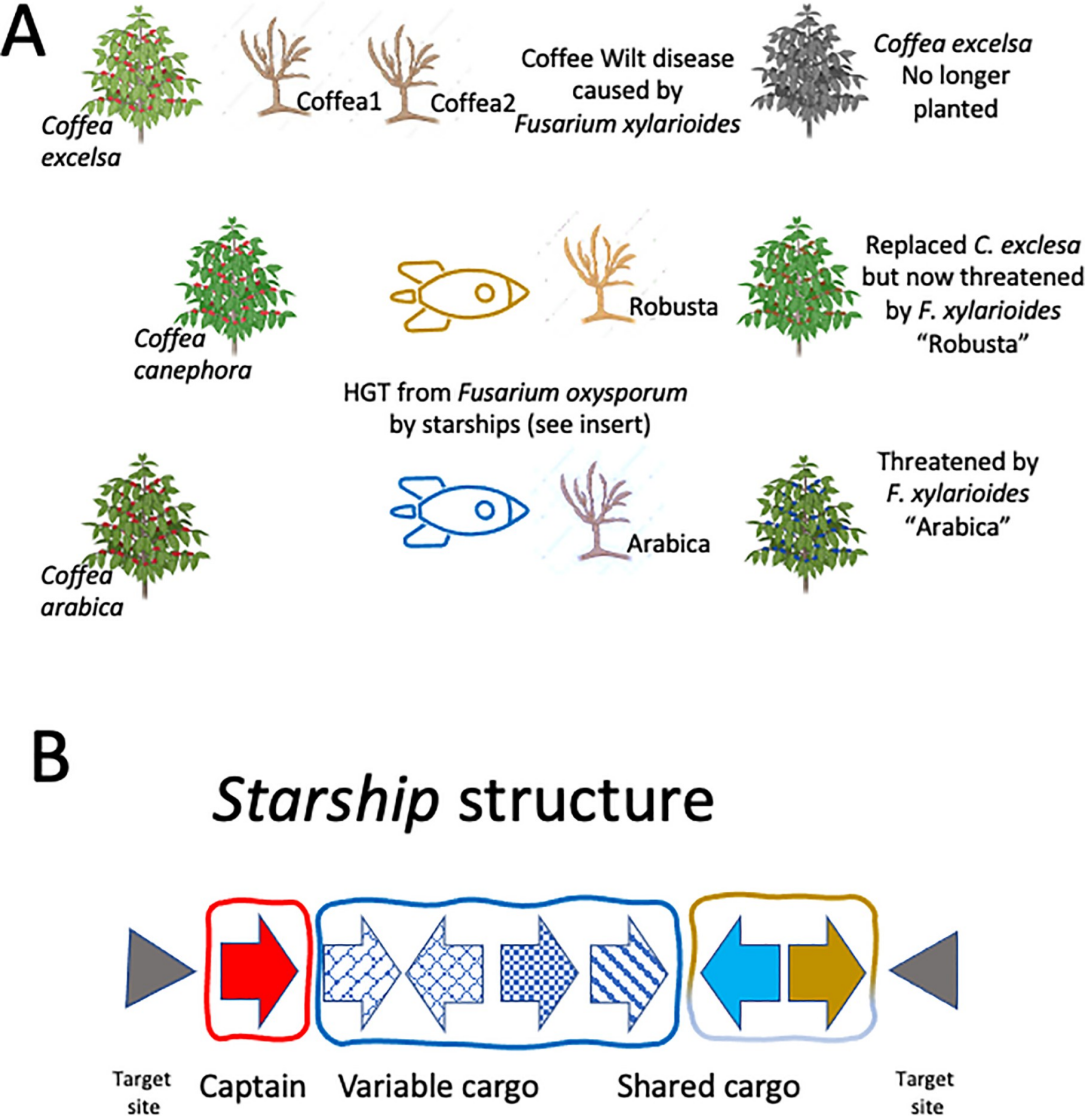
The role of horizontal gene transfer and Starships in coffee wilt disease. **(A)** Until the mid-1900s, coffee production in Africa was predominantly based on *Coffea excelsa* and *Coffea arabica*. However, the devastating impact of CWD caused by the fungal pathogen *Fusarium xylarioides*, led to the replacement of *C*. *excelsa* by *C*. *canephora* (also known as *C*. *robusta*) in many regions. The study by Peck and colleagues [3] in *PLOS Biology* on historical isolates of the CWD pathogen showed that there are at least 4 distinct lineages of this pathogen, including “Robusta” and “Arabica” that are host-specific and 2 others named “Coffea 1” and “Coffea 2.” Their study suggests that the “Robusta” and “Arabica” lineages arose through HGT facilitated by so-called “Starships” in 2 independent events. These new pathogen lineages threaten future coffee production in Africa and potentially globally. **(B)** Proposed Starship structure. The Captain gene (red), a putative Tyrosine Recombinase (examples HhpA and KIRC) has a conserved location and orientation. There is a consensus target site (gray triangle) of TTAC where the starship integrates into the genome. Several starships have been identified and while some share genes (shared cargo), there are others with highly variable (variable cargo) numbers and types of genes.

There are 2 recognized host-specific strain types of *F*. *xylarioides;* one that infects *C*. *arabica* in Ethiopia, and the other causing disease on *C*. *robusta* in Uganda, the Democratic Republic of Congo, and northern Tanzania. A recent study published in *PLOS Biology* by Peck and colleagues [3], used comparative genomic analysis of historical *F*. *xylarioides* isolates to reveal the existence of at least 4 distinct strain types of the pathogen. Two of these strain types designated “Arabica” and “Robusta” are host-specific, infecting *C*. *arabica* and *C*. *canephora*, respectively (Fig 1A). Managing future CWD outbreaks and safeguarding coffee production globally will depend on understanding these dynamics relating to the genetics and biology of *F*. *xylarioides*.

Unlike most multicellular eukaryotes, fungi lack specialized organs that protect their germline. This means that fungal cells are continuously exposed to foreign DNA from other organisms that co-occur in their environment. Because every cell within a fungal mycelium has the potential to undergo cell division, any horizontally transferred genes could be incorporated into the genome, replicated, and passed on to future generations. This makes it possible for fungi to more readily acquire foreign genetic material than other multicellular organisms with more rigidly protected germlines. The new study by Peck and colleagues [3] highlights the potential role of horizontal gene transfer (HGT) in the repeated emergence of CWD over the last century. This genetic exchange is thought to have contributed to the adaptation and persistence of the pathogen, allowing it to infect different species of *Coffea* and thus to sustain outbreaks across Africa. The study of Peck and colleagues [3] in *PLOS Biology* adds new knowledge to our understanding of this important process.

HGT is well documented in prokaryotes (Bacteria and Archaea), with numerous underlying mechanisms having been described [4]. Such exchange of genetic material enables bacteria to rapidly acquire new traits, promoting genetic diversity and adaptation, contributing to the emergence of drug-resistant strains and enhancing their ability to thrive in changing environments. Understanding how these processes function has been fundamentally important in developing strategies to combat antibiotic resistance and bacterial pathogenicity. In contrast, HGT is much less-well understood in eukaryotes including fungi.

The increasing availability of accessible whole-genome sequencing is rapidly making it possible to understand HGT in fungi. Long-read sequencing technologies, which allow for assemblies across repetitive sequences, make it possible to produce more contiguous and even chromosome level genome assemblies, allowing researchers to track the movement and associations of mobile genetic elements. This is especially true for fungi due to their relatively small genomes. It is consequently feasible to compare the complete genome sequences of hundreds of species and thus to reveal patterns of HGT and the role of mobile genetic elements in shaping genetic diversity.

Recently, researchers have reported the insertion of unusually large DNA segments into fungal genomes [5,6]. These segments, termed “starships,” represent a newly discovered category of massive mobile elements, averaging about 110 kb in length. Starships are characterized by their conserved components and the presence of a diverse array of accessory genes (Fig 1B). In keeping with their science fiction-inspired nomenclature, starships include a gene termed “Captain” that encodes a tyrosine recombinase. This enzyme is crucial for the mobilization of starships, guiding their insertion into specific genomic locations with a target site consensus sequence. The remaining genes within starships are referred to as “cargo” reflecting their role in carrying additional genetic material. Starships are proposed to be the eukaryotic analogues of bacterial integrative and conjugative elements [7] and it is suggested that they represent a dedicated mechanism of active gene transfer in eukaryotes and that they could be involved in the birth and/or growth of accessory chromosomes [5].

The study of Peck and colleagues [3] in *PLOS Biology* adds to a growing number of examples where large fragment HGT has been shown in fungi. The authors demonstrate, using comparative genomics (and transcriptomics), that large genomic regions have been acquired by isolates of the coffee wilt fungus *Fusarium xylarioides* [1] from the distantly related fungus *F*. *oxysporum*. The acquired genes are homologues of those reported from a mobile pathogenicity chromosome in *F*. *oxysporum* [8,9] and have likely contributed to host specificity in *F*. *xylarioides*. The authors hypothesize that HGT in the form of a Starship has contributed to the repeated emergence of coffee wilt disease across Africa.

When examining HGT and the newly discovered starships, it is important to recognize that closely related fungal species can hybridize, producing hybrids that contain genes from both parent species. Over successive generations very few genes may be retained from the one parent species. Differentiating between genes acquired by hybridization and those acquired though HGT such as through Starship movement is thus challenging. In addition, over evolutionary time, some genes can also be lost in certain lineages due to incomplete lineage sorting, complicating the attribution of large genetic elements solely to HGT. Starships have only recently been recognized, but several distinct types have already been identified, including Enterprise, Voyager, Argo, Phoenix, and Defiant [5]. While these are exciting new discoveries, our understanding of the role of starships in fungal genomes remains rudimentary, with many interesting questions awaiting answers.

The study of Peck and colleagues [3] focussed on the coffee wilt pathogen, provides additional evidence regarding the role that starships could play in the evolution of fungal pathogens. Their research suggests that starships may not only facilitate the movement of large segments of DNA but might also be important in HGT between species and the movement of accessory chromosomes. As investigations on this topic increase in number, more precise definitions and clearer insights into this novel mechanism of gene sharing will emerge. Ongoing research will surely also reveal the broader impacts of starships and other large mobile genetic elements on fungal genetic diversity and evolution.
